# A Retrospective Evaluation of Risk of Peripartum Cardiac Dysfunction in Survivors of Childhood, Adolescent and Young Adult Malignancies

**DOI:** 10.3390/cancers11081046

**Published:** 2019-07-24

**Authors:** Lori Chait-Rubinek, Justin A. Mariani, Natalie Goroncy, Alan Herschtal, Greg C. Wheeler, Mary K. Dwyer, John F. Seymour, Belinda A. Campbell

**Affiliations:** 1 School of Medicine, University of Notre Dame, Sydney 2010, Australia; 2 Department of Cardiovascular Medicine, Alfred Hospital, Melbourne 3000, Australia; 3 Central Clinical School, Faculty of Medicine, Monash University, Clayton 3168, Australia; 4 Heart Failure Research Group, Baker Heart and Diabetes Research Institute, Prahran 3181, Australia; 5 Department of Nursing, Peter MacCallum Cancer Centre, Melbourne 3000, Australia; 6 Centre for Biostatics and Clinical Trials, Peter MacCallum Cancer Centre, Melbourne 3000, Australia; 7 Department of Radiation Oncology, Peter MacCallum Cancer Centre, Melbourne 3000, Australia; 8 Department of Clinical Pathology, University of Melbourne, Parkville, VIC 3000, Australia; 9 Department of Clinical Haematology, Peter MacCallum Cancer Centre and Royal Melbourne Hospital, Melbourne 3000, Australia; 10 Sir Peter MacCallum Department of Oncology, University of Melbourne, Parkville, VIC 3000, Australia

**Keywords:** peripartum cardiomyopathy, anthracycline cardiotoxicity, late effects, cardiac dysfunction, radiotherapy, long-term survivors, pregnancy, malignancy

## Abstract

Long-term survivors of childhood, adolescent and young adult (AYA) malignancies with past exposure to potentially cardiotoxic treatments are at risk of peripartum cardiac dysfunction. Incidence and risk factors for peripartum cardiac dysfunction and maternal cardiac outcomes in this population were investigated. Eligible long-term survivors were aged <30 years at cancer diagnosis, with ≥1 pregnancy occurring ≥5 years after diagnosis. “Peripartum” cardiac events were defined as occurring within pregnancy or ≤5months after delivery. Cardiac events were classified “symptomatic” or “subclinical”. “Peripartum cardiomyopathy” (PPCM) was defined as symptomatic dysfunction without prior cardiac dysfunction. Of 64 eligible women, 5 (7.8%) had peripartum cardiac events: 3 symptomatic, 2 subclinical. Of 110 live births, 2 (1.8%, 95% CI 0.2–6.4) were defined as PPCM: Significantly greater than the published general population incidence of 1:3000 (*p* < 0.001), representing a 55-fold (95% CI 6.6–192.0) increased risk. Risk factor analyses were hypothesis-generating, revealing younger age at cancer diagnosis and higher anthracycline dose. Postpartum, cardiac function of 4 women (80%) failed to return to baseline. In conclusion, peripartum cardiac dysfunction is an uncommon but potentially serious complication in long-term survivors of paediatric and AYA malignancies previously treated with cardiotoxic therapies. Peripartum cardiac assessment is strongly recommended for at-risk patients.

## 1. Introduction

Long-term survivors of malignancy are at risk of late complications from exposure to past cytotoxic therapies [1]. Anthracycline-containing chemotherapy and chest radiotherapy are known to be associated with long-term, dose-dependent cardiotoxicity, which can manifest as clinical or subclinical cardiac dysfunction [2,3]. Known contributing factors to cardiotoxicity include higher cumulative anthracycline and radiotherapy doses, combined treatment modalities, younger age at treatment and female gender [2,3,4,5,6,7]. Long-term survivors of paediatric, adolescent and young adult (AYA) malignancies represent a particularly vulnerable group due to their younger age at past exposure, likelihood of poor patient recollection of past treatments and potential patient phobias stemming from these early medical interactions at a younger age [8].

Due to improved cure rates and fertility preservation, more female survivors of childhood and AYA malignancies are now reaching adulthood with reproductive potential. For the recipients of past cardiotoxic therapies, peripartum cardiac dysfunction is a concern [9,10], in particular, the ability to accommodate the associated physiological cardiovascular demands of pregnancy and childbirth, including increased maternal blood volume, preload, heart rate and cardiac output [11,12].

Within the general population, ‘peripartum cardiomyopathy’ (PPCM) is a rare condition with an estimated incidence of 1 in 3000 live births [11,12]. PPCM is typically a dilated cardiomyopathy, defined as heart failure during pregnancy (usually the last trimester) or ≤5 months postpartum [11,12]. However, there is limited data regarding the comparative cumulative incidences of pregnancy-related cardiac events in long-term survivors of malignancy. Furthermore, the risk factors for peripartum cardiac dysfunction in long-term survivors of malignancy are unknown. 

In this study, we investigate the risk of peripartum cardiac dysfunction in long-term survivors of malignancy. We describe these cardiac events, explore risk factors and assess postpartum cardiac recovery.

## 2. Results

### 2.1. Patient Characteristics 

**Figure 1:**
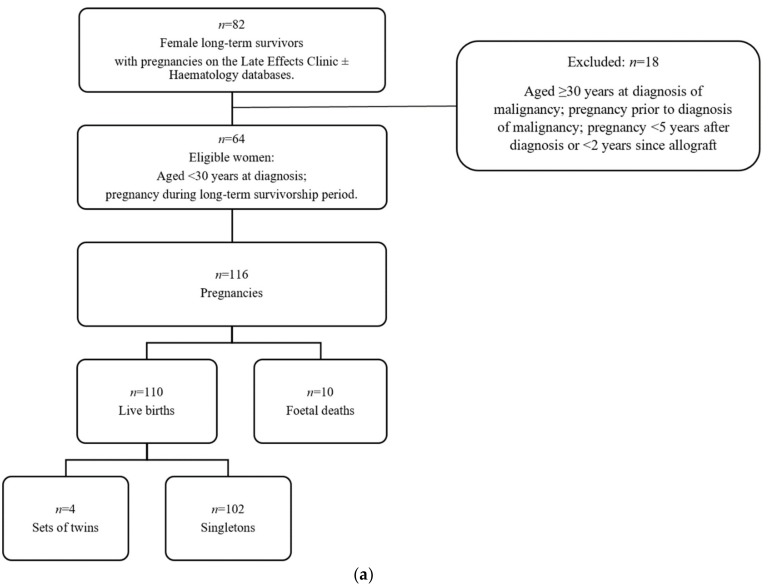

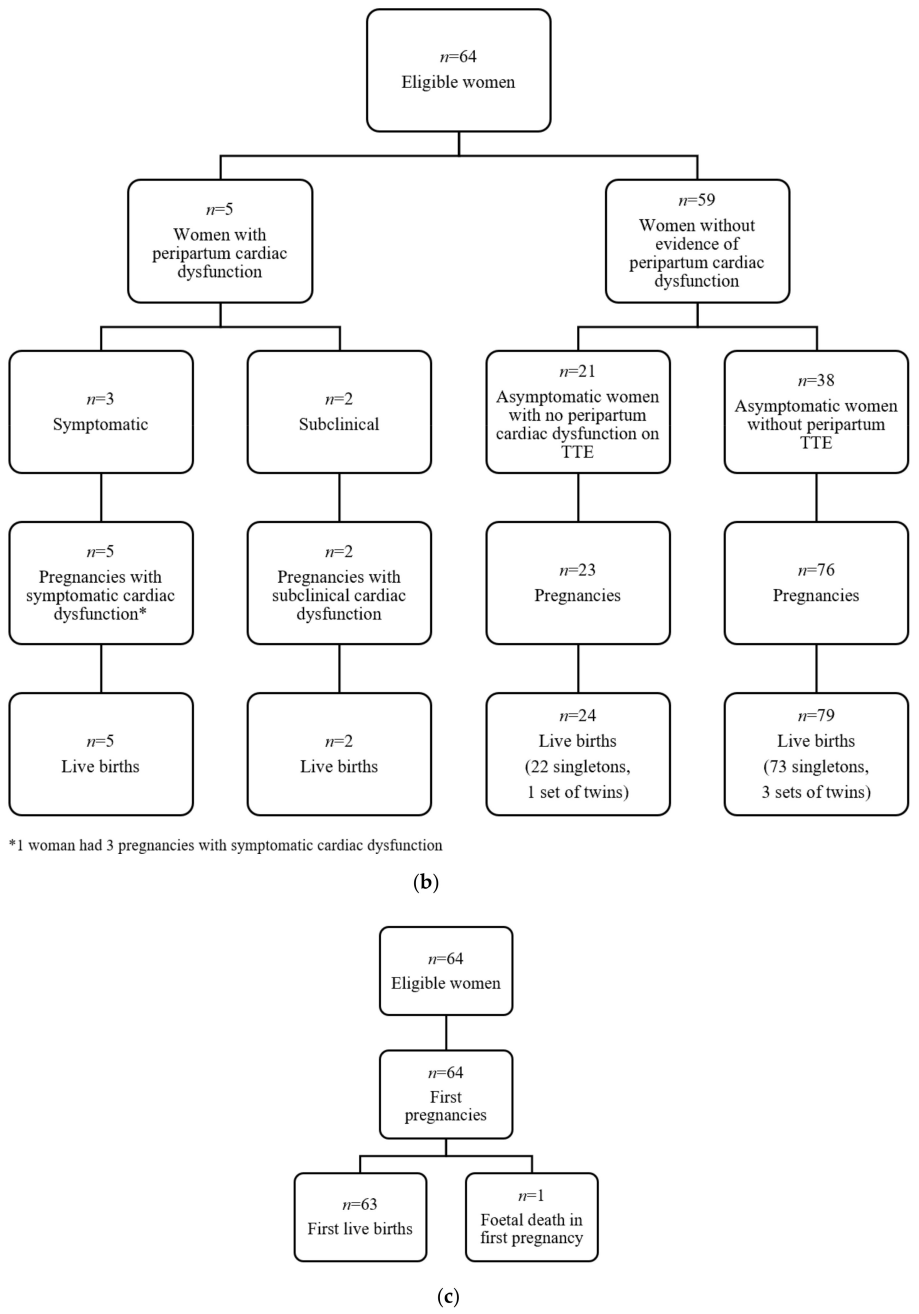
Patient eligibility. (**a**) Per “live birth” (*n* = 110); (**b**) Per “patient” (*n* = 64); (**c**) Per “first live birth” (*n* = 63).

Sixty-four female, long-term survivors were eligible (Figure 1a). Clinical characteristics are described in Table 1. Median age at diagnosis of malignancy was 18 years (range 2–29). Fifty-five women (86%) previously received anthracycline chemotherapy: 41 received ≤300 mg/m^2^, 13 received >300 mg/m^2^ and 1 unknown. The median anthracycline dose for solid tumours was greater than for haematological malignancies (337.5 mg/m^2^ vs. 250 mg/m^2^). Thirty-seven women (58%) had previously received chest radiotherapy, median dose 36 Gy (range 12–50 Gy). Twenty-eight women (44%) had received both anthracyclines and chest radiotherapy.

### 2.2. Characteristics of Pregnancies and Live Births 

In total, 116 pregnancies were identified (Figure 1a), with 110 live births (102 singletons and 4 sets of twins). There were 10 foetal deaths: 7 spontaneous miscarriages, 2 induced terminations of pregnancy and 1 ectopic pregnancy. Of note, none of the foetal deaths were attributable to maternal cardiac dysfunction.

The median time from diagnosis of malignancy to first pregnancy was 11 years (range 5–37 years) and median maternal age at first pregnancy was 31 years (range 19–42), (Table 1). There were no cases of neonatal or maternal mortality. Two of the five women with cardiac dysfunction delivered prematurely (<37 weeks gestation). 

### 2.3. Characteristics of Cardiac Events 

All women with peripartum cardiac dysfunction had received anthracycline chemotherapy, with median equivalent cumulative dose of 360 mg/m^2^ (range 280–480). None had received chest radiotherapy (Table 2). 

### 2.4. Symptomatic Cardiac Events

In this cohort (*n* = 64), 3 women (4.7%, 95% CI 1.0%, 13.1%) experienced symptomatic cardiac events in the peripartum period (Figure 1b).

Patient 1 was diagnosed with Ewing sarcoma at age 9 years; treatment included doxorubicin (cumulative dose of 480 mg/m^2^). Pre-pregnancy, cardiac comorbidities included mild hypertension and dyslipidaemia. Natural conception occurred at age 37 years: 28 years after sarcoma diagnosis. Pre-pregnancy, transthoracic echocardiography (TTE) was performed at 22, 25 and 28 years post-cancer treatment (LVEF 55%, 53%, 63%, respectively). The second of these TTE studies also demonstrated mild atrial dilation; however, the patient was asymptomatic. Screening TTE at 20 weeks gestation demonstrated a low-normal LVEF (53%). At 34 weeks gestation, the patient was diagnosed with HELLP (haemolysis, elevated liver enzymes, low platelet count) syndrome, necessitating emergency caesarean section. Four months postpartum, the patient presented with shortness of breath and decreased exercise tolerance, rapidly progressing to severe decompensated heart failure (NYHA Grade 4) with pulmonary oedema. TTE revealed a drop in LVEF to 20% plus evidence of bi-atrial and left ventricular enlargement. She was admitted to the coronary care unit and responded to diuresis and anti-failure medications. Eighteen months postpartum, her cardiac function remained low (LVEF 35%). The most recent cardiac magnetic resonance imaging (MRI) (3 years postpartum), demonstrates ongoing failure to return to baseline with left ventricular dilation and no evidence of fibrosis (LVEF 52%).

Patient 2 was diagnosed with hepatoblastoma at age 4 years and received doxorubicin (360 mg/m^2^ total cumulative dose). Pre-pregnancy, other cardiac risk factors included cigarette smoking and strong family history of ischaemic heart disease. Maternal age was 22 years at first pregnancy, 18 years after her previous cancer diagnosis. TTE was within normal limits 10 months prior to pregnancy (LVEF 55%) and at 26 weeks gestation (LVEF 60%). At 36 weeks gestation, she developed symptomatic flash pulmonary oedema, presenting with severe shortness of breath. TTE demonstrated LVEF < 15% with mild left atrial dilation. Emergency induction of labour and a vaginal instrumental delivery (forceps) took place within the intensive care unit with cardiac monitoring. During the 5 months postpartum period, her LVEF remained low (15–35%); later improving to a below-baseline level of 49% at 6 months post-delivery. The patient was advised to undergo a tubal ligation to avoid the cardiac morbidity/mortality risks from future pregnancy. 

Patient 3 was diagnosed with Ewing Sarcoma of the vertebrae at age 13 years and received doxorubicin (280 mg/m^2^ cumulative dose). She had no other known cardiac risk factors. Pre-pregnancy baseline TTE demonstrated a FS measurement of 50%. During her first pregnancy, which occurred six years after sarcoma diagnosis, she experienced palpitations. TTE revealed an impaired systolic function (FS 21%) requiring anti-failure medication. TTE performed three years post pregnancy demonstrated ongoing below-baseline heart function (FS 24%). This patient subsequently had two further pregnancies. In both she experienced symptoms of heart failure correlating with systolic dysfunction evident on TTE (FS 22% and LVEF 46%). All three pregnancies led to vaginal deliveries, between 37–40 weeks gestation. The first pregnancy was complicated by pre-eclampsia and the third was complicated by premature rupture of membranes. 

### 2.5. Subclinical Cardiac Events 

Two of 64 patients (3.1%, 95% CI 0.4–10.8%) were found to have asymptomatic peripartum cardiac dysfunction (Figure 1b).

Patient 4 was diagnosed with Ewing sarcoma at age 12. She received doxorubicin (440 mg/m^2^ cumulative dose) and cyclophosphamide (2.4 g/m^2^). On completion of oncological treatment, FS was normal on TTE (35%). Pre-pregnancy, she had no other known cardiac risk factors. Maternal age at first pregnancy was 26 years, 14 years after initial sarcoma diagnosis. Asymptomatic cardiac dysfunction was detected on screening TTE in her first pregnancy: FS measured 25% in the second trimester and 22% in the third trimester. Delivery was at 37 weeks via caesarean section. Her next available TTE was 4 years later, approximately 10 months after her second pregnancy, demonstrating ongoing below-baseline FS of 27%. 

At age 14, Patient 5 was diagnosed with classical Hodgkin lymphoma and received epirubicin (450 mg/m^2^ cumulative dose, 301.5 mg/m^2^ equivalent cumulative dose). Prior to pregnancy, she had no other known cardiac risk factors and a normal baseline TTE demonstrating an LVEF of 55%. First pregnancy occurred 17 years after initial lymphoma diagnosis at age 31 years, ending in spontaneous abortion with no known peripartum cardiac event. Second pregnancy occurred 22 years after initial lymphoma diagnosis at age 36. Cardiac function was assessed 14 months prior to second pregnancy with normal systolic baseline (LVEF 62%). During pregnancy, a screening TTE was performed in accordance with the policy of the Late Effects Clinic. At 14 weeks gestation, LVEF was within normal range (56%). However, this subsequently dropped to 44% at 35 weeks gestation leading to hospitalisation for cardiac monitoring. At 36 weeks gestation, the LVEF spontaneously improved to 58%. At 41 weeks gestation the patient underwent an instrumental delivery, complicated by neonatal shoulder dystocia. Three months post-delivery, postpartum TTE demonstrated a return to normal baseline systolic function (LVEF 63%).

### 2.6. Risk Factors for Cardiac Dysfunction and Symptomatic Cardiac Dysfunction Per First Live-Birth 

Of the 63 first live births, 20 (32%) had peripartum TTE and were included in the analyses for cardiac dysfunction (Table 3) (Figure 1c).

On univariate analysis, greater anthracycline dosage (*p* = 0.015) and younger age at cancer diagnosis (*p* = 0.031) were statistically significant predictors for cardiac dysfunction. 

Past diagnosis of solid tumour was the sole significant predictor of symptomatic cardiac dysfunction (*p* = 0.009, Fisher’s exact test).

A sensitivity analysis was performed considering all live births (*n* = 110) of which 31 (28%) had peripartum TTE, which were included in the analyses for cardiac dysfunction. Results were supportive of the main analysis (Supplementary Materials).

### 2.7. Incidence of PPCM Per Live Birth and Comparison with the General Population

Of the 110 live births, two women with one live birth each (1.8%, 95% CI 0.2–6.4) had cardiac dysfunction that fulfilled the strict criteria for PPCM. In the published literature, the estimated prevalence of PPCM in the general population is reported to be 1 in 3000 [11,12]. In the present study, the rate of PPCM in long-term survivors of malignancy was significantly greater than the estimated incidence of PPCM reported in the general population (1.8% vs. 0.033%; *p* < 0.001), with relative risk estimated to be approximately 55 times greater in this patient cohort than the general population (95% CI 6.6–192.0).

### 2.8. Maternal Outcomes Beyond the Postpartum Period

#### 2.8.1. Outcomes for Women with Cardiac Events (n = 5) 

**Figure 2:**
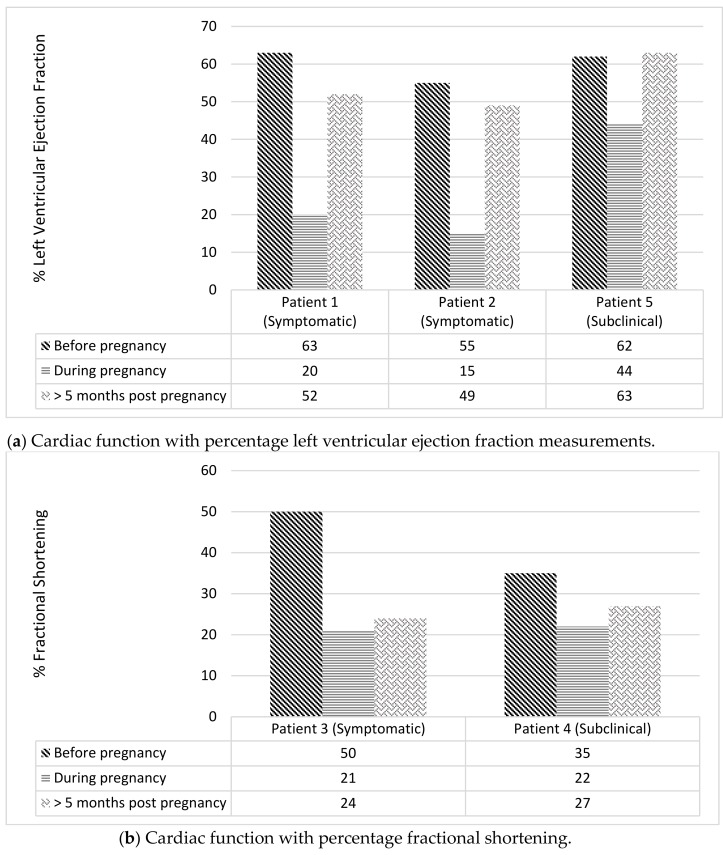
Cardiac function on transthoracic echocardiography and cardiac MRI (i) pre-, (ii) during and (iii) post-pregnancy in long-term survivors of malignancy with cardiac events.

The results of the TTE are demonstrated in Figure 2. Postpartum cardiac function failed to return to baseline in four of the five women (80%): three women with symptomatic heart failure remain on medication and receive long-term cardiology follow-up, the fourth patient died of a new malignancy. In the fifth woman with documented peripartum cardiac dysfunction, the LVEF returned to baseline shortly before childbirth and her cardiac function remained normal throughout the postpartum period and beyond. 

#### 2.8.2. Outcomes for Women with No Cardiac Events (*n* = 59)

Fifty-nine women had no reported cardiac events occurring in the defined peripartum period. Of these, four (7%) had subsequent diagnoses of A-CHF beyond the postpartum period and one (2%) had a diagnosis of A-CHF prior to pregnancy. In this latter patient, baseline FS was 16–24% prior to pregnancy; however, the pregnancy was clinically uneventful with an unexplained recovery in LVEF (63%) postpartum.

## 3. Materials and Methods 

### 3.1. Patient Selection and Study Design

This retrospective study was approved by the Peter MacCallum Cancer Centre Ethics Committee. Eligible patients were identified from the Peter MacCallum Cancer Centre Late Effects Clinic (LEC) and Haematology databases. Eligibility included long-term survivors of childhood/AYA malignancies, aged <30 years at the time of oncological diagnosis [13], with subsequent pregnancies. “Long-term survivorship” was defined as survival ≥5 years following the most recent oncological treatment, or ≥2 years post allogenic stem cell transplantation. Exclusion criteria included pregnancies occurring prior to the long-term survivorship time point. For eligible women who were diagnosed and treated for malignancy after their pregnancies, the subsequent oncological treatment and cardiac data were excluded from the analysis.

The records of eligible patients were retrospectively reviewed for information regarding demographics, past oncological diagnoses and past oncological treatments including equivalent cumulative anthracycline doses (calculated by multiplying doxorubicin × 1, daunorubicin × 0.833, epirubicin × 0.67, idarubicin × 5 and mitoxantrone × 4) [14]. Additionally, pre-existing cardiac risk factors and obstetric history were obtained. TTE and cardiac MRI from the antenatal, perinatal and postnatal periods were reviewed and details of patient management and clinical outcomes were recorded. 

### 3.2. Definitions of Cardiac Dysfunction

Symptomatic cardiac dysfunction during pregnancy: The presence of symptoms or signs of congestive heart failure occurring at any stage within pregnancy or ≤5 months post-delivery. It was defined and measured according to:

(a) Clinical features: Measured by the New York Heart Association (NYHA) Functional Classification [15] with demonstration of specific clinical signs of cardiac decompensation: pulmonary oedema (radiographic or clinical), auscultation of S3, description of orthopnoea or paroxysmal nocturnal dyspnoea and/or response to diuretic therapy.
(b) Pharmacological treatments: Requiring anti-failure therapy or diuretic therapy.
(c) Functional quantification: Abnormal systolic function defined as FS < 28% or LVEF < 50%, as evidenced on TTE or cardiac MRI.

Subclinical cardiac dysfunction during pregnancy was defined as an absence of the above clinical features with abnormal cardiac function (FS < 28%, LVEF < 50%) during pregnancy or ≤5 months post-delivery.

“Peripartum cardiomyopathy” or “PPCM” is a pre-defined diagnosis that requires pre-pregnancy cardiac assessment [11]. To meet this diagnosis, patients with symptomatic heart failure during pregnancy were required to have had pre-pregnancy TTE confirming the absence of pre-existing cardiac dysfunction. This definition has been previously reported in the general obstetric population with an estimated prevalence of 1 in 3000 live births [11,12].

All cardiac events were centrally reviewed by an experienced cardiologist (JM). Of note, these peripartum cardiac events are considered distinct from non-pregnancy related anthracycline-induced cardiotoxicity (A-CHF).

### 3.3. Statistical Analysis

Statistical methods consisted of standard reporting of descriptive baseline statistics carried out using the base package of the R language for statistical computing [16]. Exact logistic regression was used to test for a relationship between cardiac dysfunction (symptomatic or subclinical) and the following putative predictors; (a) age at diagnosis, (b) anthracycline dose, (c) maternal age at pregnancy and (d) interval between diagnosis and pregnancy. Fisher’s exact test was used to test for a relationship between cardiac dysfunction and dichotomous predictors including cancer type (haematological malignancy versus solid tumour) and radiotherapy treatment. Odds ratios and 95% CIs were calculated, as was the p-value for a hypothesis test with null hypothesis of no effect. *p* < 0.05 was considered statistically significant.

Modelling considered live births only to ensure an objective denominator, not confused by unreported terminations, or unknown pregnancies with spontaneous abortions. Some of the putative predictor variables were defined at the woman level (age at cancer diagnosis, anthracycline dose and cancer type), whereas others were defined at the live birth level (interval between diagnosis and pregnancy and maternal age at pregnancy). Thirty-three women in the dataset had multiple live births. The main analysis was performed considering the first live birth per woman only (*n* = 63) in order to remove any nesting due to similarity between live births within the same woman. One woman had two pregnancies with zero live births, which were excluded from analyses. A sensitivity analysis was also performed considering all live births (*n* = 110). Incidence calculations included all live births. An exact method for estimating proportions (Clopper-Pearson) was used to estimate the incidence of pregnancy related cardiac dysfunction amongst all live births. This was compared with that of the general population published figure of 1 in 3000 live births [11,12]. Only women with ≥1 TTE during pregnancy or within 5 months postpartum were included in the analyses for cardiac dysfunction.

## 4. Discussion

The LEC at the Peter MacCallum Cancer Centre offers a dedicated, multi-disciplinary, risk-based, screening programme for long-term survivors of childhood and AYA cancers treated with cytotoxic therapies [17]. For those who have received cardiotoxic treatments, long-term care is multifaceted: (1) screening of cardiac function, (2) minimising modifiable cardiovascular risk factors, (3) promoting primary cardiovascular health care and (4) specialist cardiology care if deterioration of cardiac function is detected or if pregnancy is planned. The LEC program recommends surveillance of cardiac function before, during and after pregnancy for all women with prior exposure to potentially cardiotoxic therapies, with coordinated care from a specialised cardiologist. Outside of LEC, however, long-term survivors of childhood and AYA malignancies may potentially be overlooked as a high-risk group for pregnancy-related cardiac dysfunction. In this retrospective analysis of the LEC and Haematology databases, only 32% of women had screening TTE prior to their first live birth, potentially reflecting a poor appreciation in the wider medical community regarding the increased cardiac risks facing these patients.

The results of this study suggest an increased risk for PPCM in this cohort of long-term survivors of paediatric and AYA malignancies. Although the absolute risk of PPCM was still found to be low (1.8%), it is significantly greater than the reported background risk of 1 in 3000 in the general population (*p* < 0.001) [11,12]. Our results represent an estimated 55-fold (95% CI 6.6–192.0) increased risk of PPCM in this cohort of long-term survivors. This finding strongly emphasises the importance of peripartum cardiac surveillance in long-term survivors of malignancy with prior exposure to cardiotoxic therapies.

During pregnancy, a lack of cardiac symptomatology does not necessarily imply cardiac well-being. This study has demonstrated that pregnancy may be the first unmasking of subclinical cardiac dysfunction in women with prior exposure to cardiotoxic therapies. This is a critically important finding for the wider medical community. Subclinical cardiac dysfunction screening enables early intervention before risking symptomatic deterioration [18,19]. Our policy and practice for early cardiology intervention is to avoid further deterioration in cardiac function, thus mitigating the risks of cardiac morbidity and mortality in the peripartum period and beyond.

The incidence of peripartum cardiac dysfunction reported in this study is higher than other previously published reports. One retrospective series from St Jude’s Children’s Research Hospital of 847 long-term cancer survivors, reported three cases of PPCM occurring within 1554 completed pregnancies, giving an incidence of 0.2%; however, this population also included 222 (27%) patients who had not been exposed to cardiotoxic therapies [18]. A second retrospective series investigated the development of symptomatic peripartum cardiac dysfunction in 53 female childhood cancer survivors previously treated with anthracyclines (mean cumulative dose 267 mg/m^2^). None of these women developed symptomatic heart failure during the peripartum period (95% CI 0–5.7%) [20]. In the present study, we identified five women with peripartum cardiac dysfunction in a cohort of 64 long-term survivors of paediatric and AYA malignancies with prior exposure to cardiotoxic therapies: three women (five pregnancies) experienced symptomatic cardiac events, two had subclinical cardiac dysfunction within the peripartum period. These studies highlight an increased risk of peripartum cardiac dysfunction for long-term survivors of malignancy previously treated with potentially cardiotoxic therapies.

Additionally, this is the first published study to investigate risk factors for peripartum cardiac dysfunction in long-term survivors of malignancy. Within the main analyses considering first live births only, higher anthracycline doses and a younger age at diagnosis were found to be associated with cardiac dysfunction. Currently, the published Children’s Oncology Group (COG) Guidelines for surveillance during pregnancy recognise a cardiac risk associated with greater anthracycline exposures; however, there is no consideration of age at the time of exposure [1]. Based on our findings, we advocate for extra vigilance in cardiac screening in pregnant long-term survivors who were younger at the time of original cancer diagnosis and/or received high anthracycline doses. 

Non-pregnancy-related A-CHF is a distinct diagnostic entity from peripartum cardiac dysfunction, but we acknowledge that there is potential for cross-over and interaction of these physiological mechanisms. In our study, the two aforementioned risk factors for peripartum cardiac dysfunction are also established risk factors for A-CHF in survivors of childhood and adolescent malignancies [6,9] and it is possible that more detailed cardiac stress testing prior to pregnancy may have unveiled subclinical cardiac impairment. Pregnancy aside, A-CHF is characterised by a continuum of cardiac dysfunction: Beginning with subclinical myocardial injury associated with asymptomatic decline in cardiac function, progressing to overt heart failure when untreated [2]. Patient 1 in our study developed overt cardiac failure during pregnancy with previous demonstrable subclinical cardiac dysfunction on TTE prior to pregnancy. It could be hypothesised that the additional cardiovascular burden of pregnancy unmasked an underlying asymptomatic A-CHF. This is supported by previous studies, which have shown that women with prior subclinical cardiac dysfunction may experience further decline during pregnancy [18,19]. In the present study, the clinical course of Patient 1’s cardiac function demonstrates that subclinical dysfunction may first become symptomatic during the peripartum period. This supports our recommendation for peripartum cardiac assessment in asymptomatic long-term cancer survivors with prior exposure to cardiotoxic therapies.

Traditionally, chest radiotherapy has been widely recognised as a risk factor for cardiac dysfunction in long-term survivors [3]. However, in the current study plus other studies, all peripartum cardiac events occurred following treatment with anthracyclines alone, and without exposure to chest radiotherapy [18]. The current COG Guidelines recommend cardiac surveillance following lower anthracycline doses only if the survivor also received some cardiac irradiation, suggesting that the additional impact of radiotherapy increases the risk of cardiac events to be equivalent to that caused by higher doses of anthracyclines alone [1]. However, whilst combination therapy is a risk factor for A-CHF [21], it may not contribute to the overall peripartum risk of cardiac events in this population. Future studies are needed to establish these risk factors and ensure that peripartum surveillance guidelines are appropriate.

This study also demonstrates the risk for long-term cardiac sequalae beyond the peripartum period, in long-term survivors of paediatric and AYA malignancies with prior exposure to cardiotoxic therapies. In this study, the cardiac function in four of five women (80%) with peripartum cardiac events failed to return to baseline following the postpartum period. This finding suggests that peripartum cardiac dysfunction may lead to long-term cardiac morbidity. We suggest that the potential seriousness of this event and the possibility of life-long cardiac morbidity indicates the need for early multi-disciplinary involvement and long-term cardiac surveillance for these high-risk women.

This study is limited by its retrospective design which created practical barriers including missing data and potential underestimation of subclinical events. Furthermore, we acknowledge the restricted cohort size and low event number as additional limitations. The lack of a matched-pair control group prevents more accurate determination of the relative increased risk of peripartum cardiac dysfunction. Therefore, we have referred to previously published data to estimate the PPCM prevalence in the general population. Moreover, the setting of the LEC represents a selected and potentially non-representative subset of the total at-risk population, as participation in this surveillance programme relies on referrals by general practitioners, specialists or self-referrals. Thus, the present study is not a true population-based cohort study, which may also impact on the accuracy of incidence calculations as well as account for the higher proportion of anthracycline treated patients in this cohort. Finally, inter-operator variability of TTE may potentially impact on the reproducibility and validity of comparing reports from different operators. Considering these limitations, risk factor analyses in this study should be considered hypothesis-generating. Larger multi-centre trials, and/or population-based screening programs are required for more rigorous analyses of incidence and risk factors for peripartum cardiac events in this at-risk population of long-term survivors.

## 5. Conclusions

Overall, peripartum cardiac dysfunction is uncommon in long-term survivors of malignancy with prior exposure to cardiotoxic therapies. However, this study has shown that the relative risk is approximately 55 times greater than the general population. This finding is of critical clinical importance for this high-risk population and their treating physicians, and long-term survivors of paediatric and AYA malignancies with prior exposure to cardiotoxic therapies should be managed cautiously with careful cardiac surveillance during the peripartum period. Current COG Guidelines for cardiac surveillance in this at-risk population recommend surveillance in women with prior exposure to significant doses of anthracyclines and/or chest radiotherapy. We suggest that particular vigilance be given to women who received higher anthracycline dose or were younger at the time of original cancer diagnosis. For pregnant long-term survivors of malignancy, risk-adjusted cardiac surveillance programs are required for optimal perinatal care. Furthermore, peripartum cardiac dysfunction may be associated with long-term cardiac morbidity and is an indication for specialist cardiology follow-up. Larger studies are required to more rigorously evaluate risk factors to enable women to make informed decisions regarding maternal cardiac safety in pregnancy.

## Figures and Tables

**Table 1:** Patient characteristics of 64 long-term survivors with subsequent pregnancies.

Clinical Characteristics	*n* = 64
**Age at diagnosis of malignancy (years)**	
Median, (Range)	18 (2–29)
**Malignancy Type (n, %)**	
Leukaemia	8 (12.5)
(a) ALL*	4 (6)
(b) AML*	3 (5)
(c) Dual phenotypes	1 (2)
Lymphoma	42 (66)
(a) NHL*	10 (16)
(b) HL*	32 (50)
Osteosarcoma	1 (1.5)
Ewing sarcoma	5 (8)
Hepatoblastoma	1 (1.5)
Wilm’s Tumour	4 (6)
Other solid tumour	3 (5)
**Anti-malignancy therapies (n, %)**	
Anthracycline chemotherapy	55 (86)
Non-anthracycline based treatment	9 (14)
Chest radiotherapy	37 (58)
(a) Chest radiotherapy only	5 (8)
(b) Anthracyclines & chest radiotherapy	28 (44)
(c) Chest radiotherapy & non-anthracycline chemotherapy	4 (6)
**Cumulative anthracycline dose (mg/m^2^)**	*n* = 55
Median, (Range)	270 (150–600)
≤300 mg/m^2^ (n, %)	41 (74)
>300 mg/m^2^ (n, %)	13 (24)
Unknown	1 (2)
**Total chest radiotherapy dose (Gy)**	*n* = 37
Median, [Range]	36 (12–50)
**Pregnancies and live-births (n)**	
Total number of reported pregnancies	116
Live births	110
Singleton births	102
Twin births	4
**Foetal deaths (n)**	
Total	10
(a) Miscarriage	7
(b) Elective termination	2
(c) Ectopic pregnancy	1
**Time from malignancy diagnosis to first pregnancy (years)**	
Median, (Range)	11 (5–37)
Unknown	1
**Maternal age at onset of first pregnancy (years)**	
Median, (Range)	31 (19–42)
Unknown	1

* ALL = Acute lymphoblastic leukaemia, AML = Acute myeloid leukaemia, NHL = Non-Hodgkin’s lymphoma, HL = Hodgkin’s lymphoma.

**Table 2:** Clinical characteristics of patients with documented peripartum cardiac events (*n* = 5).

	Patient 1	Patient 2	Patient 3	Patient 4	Patient 5
Age at cancer diagnosis (years)	9	4	13	12	14
Cancer type	Ewing sarcoma	Hepatoblastoma	Ewing sarcoma	Ewing sarcoma	Hodgkin lymphoma
Anthracycline dose mg/m^2^	480	360	280	440	301.5
Number of pregnancies	1	1	3	2	2
Number of live births	1	1	3	2	1
Time interval between cancer diagnosis and first pregnancy (years)	28	18	6	14	17
Most recent pre-pregnancy transthoracic echocardiogram	Normal, LVEF 63%	Normal, LVEF 55%	Normal, FS 50%	Normal, FS 35%	Normal, LVEF 62%
Cardiac event	Symptomatic HF	Symptomatic HF	Symptomatic HF	Subclinical	Subclinical
Functional quantification of cardiac event	LVEF 20%	LVEF <15%	FS 21%	FS 22%	LVEF 44%

HF = heart failure, LVEF = left ventricular ejection fraction, FS = fractional shortening.

**Table 3:** Risk factors for pregnancy-related cardiac events first live births only.

Predictor	Median	Number of Cardiac Events	Odds Ratio, (95% Confidence Interval (CI))	*p*-Value
Cardiac Dysfunction (*n* = 20 †) *				
Age at diagnosis of malignancy	14.5 years	5	0.853, (0.682, 1.010) **	0.031
Anthracycline dose (×10)	300 mg/m²	5	1.150, (1.010, 1.360)	0.015
Maternal age at pregnancy	29.5 years	5	0.972, (0.803, 1.170) **	0.960
Time from cancer diagnosis to pregnancy	10.5 years	5	1.090, (0.941, 1.260) **	0.200
Cancer Type			0.140, (0.002, 1.900)	0.130
(a) Haematological malignancy (*n* = 11)		1		
(b) Solid Tumour (*n* = 9)		4		
Chest radiotherapy			0.000, (0.000, 2.390)	0.260
(a) Chest radiotherapy (*n* = 6)		0		
(b) No chest radiotherapy (*n* = 14)		5		
Symptomatic cardiac dysfunction (*n* = 63) *				
Age at diagnosis of malignancy	18 years	3	0.846, (0.685, 0.994) **	0.062
Anthracycline dose (×10)	226.5 mg/m²	3	1.070, (0.987, 1.160)	0.083
(missing *n* = 1)				
Maternal age at pregnancy	31 years	3	0.825, (0.622, 1.050) **	0.120
(missing *n* = 1)				
Time from cancer diagnosis to pregnancy	11 years	3	1.080, (0.916, 1.280) **	0.260
(missing *n* = 1)				
Cancer Type			0.000, (0.000, 0.635)	0.009
(a) Haematological malignancy (*n* = 49)		0		
(b) Solid Tumour (*n* = 14)		3		
Chest radiotherapy			0.000, (0.000, 1.760)	0.074
(a) Chest radiotherapy (*n* = 36)		0		
(b) No chest radiotherapy (*n* = 27)		3		

† Of the 63 first live births, 20 patients had peripartum TTE and were included in the analyses for cardiac dysfunction. * Exact logistic regressions and Fisher’s exact tests. ** Odds ratio calculated per year.

## Supplementary Materials

The following are available online at https://www.mdpi.com/2072-6694/11/8/1046/s1, Table S1. Sensitivity analysis of risk factors for pregnancy related cardiac events for all live births. Raw data used to calculate risk factors.
