# Supplementary material for: A Retrospective Evaluation of Risk of Peripartum Cardiac Dysfunction in Survivors of Childhood, Adolescent and Young Adult Malignancies
Source: Cancers (Basel). 2019 Jul 24;11(8):1046. doi: 10.3390/cancers11081046 (PMC6721401; doi:10.3390/cancers11081046)
Supplement: Supplementary file 1 [file cancers-11-01046-s001.zip › Table S1.pdf]

Table S1. Sensitivity analysis of risk factors for pregnancy related cardiac events for all live births

**Predictor**	**Median**	**Number of cardiac events**	**Odds ratio, [95% confidence interval (CI)]**	**p-value**
Cardiac Dysfunction (N=31†)*				
Age at diagnosis of malignancy	14 years	7	0.902, [0.784, 1.010] **	0.067
Anthracycline dose (×10) (missing n=1)	280mg/m²	7	1.070, [0.992, 1.170]	0.130
Maternal age at pregnancy	31 years	7	0.889, [0.749, 1.040] **	0.130
Time from cancer diagnosis to pregnancy	11 years	7	1.010, [0.912, 1.120] **	0.620
Cancer Type<br>(a) Haematological malignancy (n=18)<br>(b) Solid Tumour (n=13)		 1<br>6	0.075, [0.001, 0.789]	0.012
Chest radiotherapy<br>(a) Chest radiotherapy (n=10)<br>(b) No chest radiotherapy (n=21)		 0<br>7	0.000, [0.000, 1.270]	0.066
***Symptomatic cardiac dysfunction (N=110)* ***				
Age at diagnosis of malignancy	21 years	5	0.882, [0.773, 0.983] **	0.017
Anthracycline dose (×10)<br>(missing n=3)	224mg/m²	5	1.040, [0.986, 1.100]	0.400
Maternal age at pregnancy<br>(missing n=5)	31 years	5	0.761, [0.575, 0.938] **	0.006
Time from cancer diagnosis to pregnancy<br>(missing n=5)	11 years	5	1.040, [0.905, 1.160] **	0.470
Cancer Type<br>(a) Haematological malignancy (n=85)<br>(b) Solid Tumour (n=25)		 0<br>5	0.000, [0.000, 0.286]	<0.001
Chest radiotherapy<br>(a) Chest radiotherapy (n=62)<br>(b) No chest radiotherapy (n=48)		 0<br>5	0.000, [0.000, 0.806]	0.014

† Of 110 live births, 31 patients had peripartum TTE and were included in the analyses for cardiac dysfunction. *Exact logistic regressions and Fisher's exact tests. **Odds ratio calculated per year.
