# Supplementary material for: A Retrospective Evaluation of Risk of Peripartum Cardiac Dysfunction in Survivors of Childhood, Adolescent and Young Adult Malignancies
Source: Cancers (Basel). 2019 Jul 24;11(8):1046. doi: 10.3390/cancers11081046 (PMC6721401; doi:10.3390/cancers11081046)
Supplement: Supplementary file 1 [file cancers-11-01046-s001.zip › Supplementary Material Explanation text.docx]

**Supplementary Material**

Table S1 in the supplementary section provides the sensitivity analysis of risk factors for pregnancy related cardiac events for all live births.
