# Supplementary material for: A Retrospective Evaluation of Risk of Peripartum Cardiac Dysfunction in Survivors of Childhood, Adolescent and Young Adult Malignancies
Source: Cancers (Basel). 2019 Jul 24;11(8):1046. doi: 10.3390/cancers11081046 (PMC6721401; doi:10.3390/cancers11081046)
Supplement: Supplementary file 1 [file cancers-11-01046-s001.zip › Data Coding.pdf]

Data Coding: Risk Factor Analysis

1. Cardiac dysfunction
    a. 1= Yes
    b. 0= No
    c. Blank= unknown

2. Symptomatic Heart failure
    a. 1= Yes
    b. 0= No

3. Cancer Type
    a. 1= Solid malignancy
    b. 2= Haematological malignancy

4. Chest Radiotherapy
    a. 1= Yes
    b. 0= No

5. Foetal death
    a. 1= Yes
    b. 0=No
